# Case report: Sequential therapy with dupilumab and baricitinib for severe alopecia areata with atopic dermatitis in children

**DOI:** 10.3389/fimmu.2024.1395288

**Published:** 2024-06-06

**Authors:** Huijuan Fang, Fengchuan Zhang, Wenjun Lin, Yuqi Jiang, Qingwu Liu, Dingquan Yang

**Affiliations:** ^1^ Dongfang Hospital, Beijing University of Chinese Medicine, Beijing, China; ^2^ Dermatology Department, China-Japan Friendship Hospital, Beijing, China

**Keywords:** alopecia areata, atopic dermatitis, autoimmunity, baricitinib, dupilumab

## Abstract

An 8-year-old female child presented with patchy hair loss for 1 year, accompanied by eyebrow loss for 6 months. Microscopic examination of the hair confirmed the features of active stage alopecia areata, with a Severity of Alopecia Tool (SALT) score of 70%. The diagnosis was severe alopecia areata. The patient had a history of atopic dermatitis since infancy, with recurrent episodes of scattered papules and pruritus for 8 years. Initial treatment involved subcutaneous injections of dupilumab 300mg every 2 weeks for 6 months, resulting in a reduction of SALT score to 20% and improvement of atopic dermatitis symptoms. Discontinuation of Dupilumab and initiation of daily oral Baricitinib at a dose of 2mg for a duration of 5 months. According to the SALT score evaluation, the severity of hair loss was less than 10% and there was significant regrowth of hair. No significant adverse reactions were observed during the treatment period.

## Introduction

Alopecia areata (AA) is a common immune-mediated, inflammatory, non-scarring hair loss condition, with a slightly higher incidence in children compared to adults. The exact pathogenesis of AA is not fully understood, but histopathologically, lymphocytic T-cell infiltration around the hair follicles can be observed. Studies have indicated that childhood AA often has a familial predisposition, and severe cases in children can pose challenges in treatment, with possible resistance to conventional therapies (1). Immune-mediated inflammatory skin diseases encompass a group of commonly associated conditions, including atopic dermatitis (AD), vitiligo, and AA, with clinical overlap in some cases, and the severity of the diseases showing correlation, making treatment more complex (2). In this article, we present a case study involving an 8-year-old patient who had both severe alopecia areata and atopic dermatitis. The patient underwent a 6-month treatment involving Dupilumab subcutaneous injections, followed by a 5-month treatment with Baricitinib oral medication. The therapeutic outcomes were highly satisfactory, as evidenced by significant improvement in AD symptoms and substantial regrowth of hair in most of the affected areas of alopecia. The study has received approval from the Institutional Ethics Committee of China-Japan Friendship Hospital, with the assigned approval number of 2020-127-K80.

## Case report

**Figure 1:**
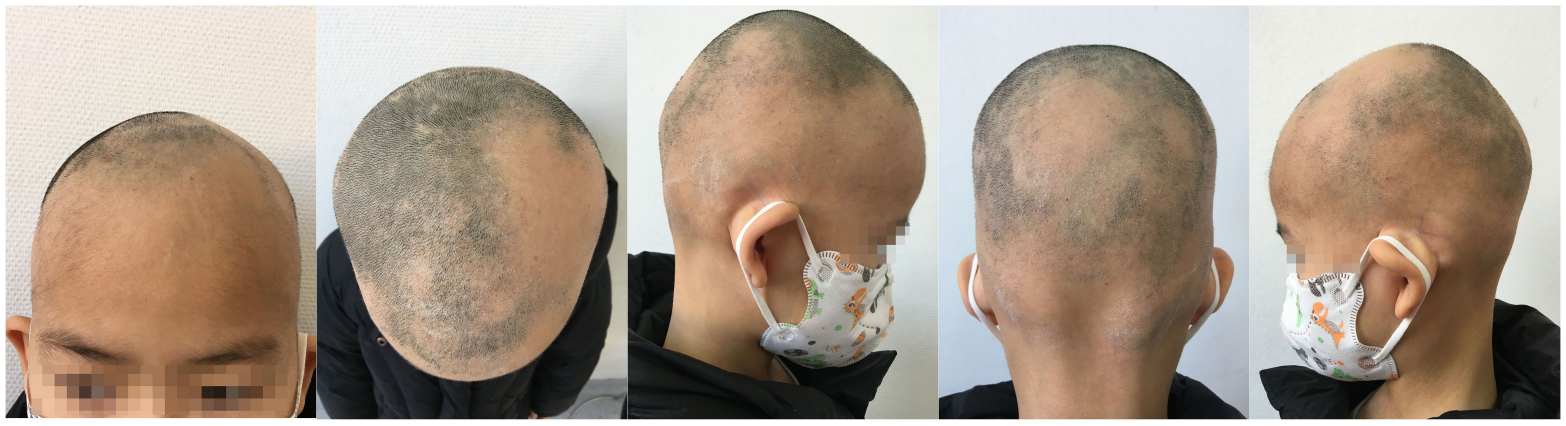
During the patient’s first visit, we noticed a significant amount of hair loss, while there were no observable changes in the eyebrows and eyelashes.

**Figure 2:**
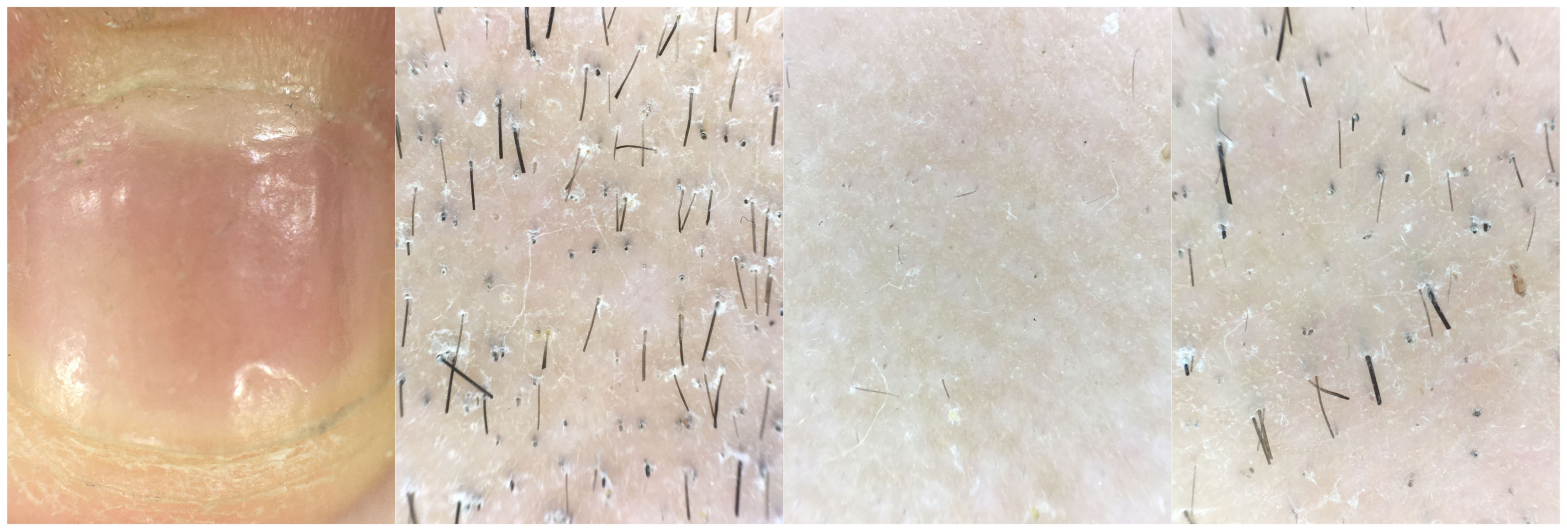
Clinical photographs reveal nail damage, while dermatoscopic examination shows the presence of black dot sign, exclamation mark hairs, and broken hairs at the site of skin lesions.

**Figure 3:**
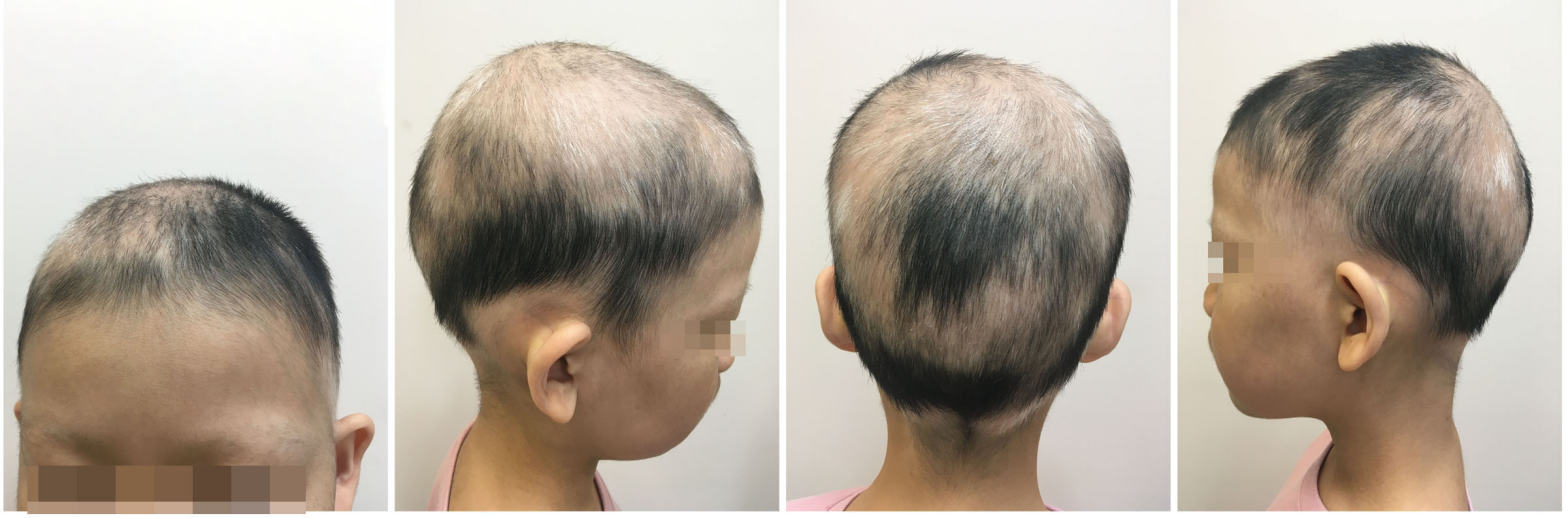
Partial hair regrowth and the presence of white hairs were observed during the second visit, but there was partial eyebrow loss.

**Figure 4:**
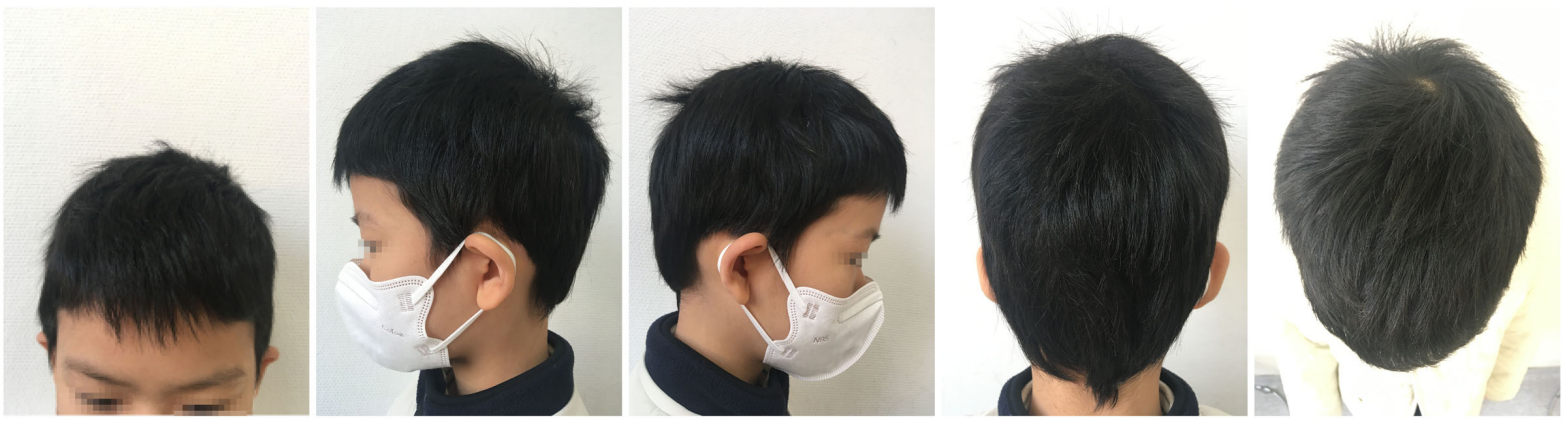
After 11 months of sequential treatment, the patient’s hair has almost completely regrown.

An 8-year-old female patient, with a height of 135cm and weight of 26.5 kilograms, presented with coin-sized patches of hair loss on the scalp. The onset occurred one year ago without any known precipitating factors. The patient had previously been diagnosed with alopecia areata and received treatment with minoxidil solution and topical clobetasol, but with no significant improvement. The patient also had a history of atopic dermatitis, which worsened during the hair loss period and was accompanied by noticeable itching. The patient’s father had a personal history of alopecia areata, but there was no significant family history of the condition. Dermatological examination showed patchy hair loss on the scalp without eyebrow or eyelash involvement (**Figure 1**). The Severity of Alopecia Tool (SALT) score was 70%. During the initial visit, the examination revealed dark red patches and pinpoint-sized papules on the buttocks, groin, and limbs. These areas exhibited lichenification, scratch marks, erosion, crusting, dryness, scaling, and significant pruritus. The Eczema Area and Severity Index (EASI) score was 50.3, the Scoring Atopic Dermatitis (SCORAD) score was 72.1, and the Children’s Dermatology Life Quality Index (CDLQI) score was 27. Laboratory findings indicated elevated levels of total immunoglobulin E (IgE) and an increased proportion of double-negative T cells (CD3+CD4-CD8-). Allergen testing revealed sensitivities to various substances, including egg white and wheat. Other laboratory tests were within normal limits. Dermoscopy revealed characteristic features such as black dots, broken hairs, and exclamation mark hairs in the affected areas. Some areas showed reduced hair follicle openings or absence of hair, with only minimal vellus hairs. Nail abnormalities were also observed (**Figure 2**). The diagnosis is severe alopecia areata. The patient received subcutaneous injections of 300mg of dupilumab every two weeks for a duration of six months. Following a six-month course of treatment with dupilumab, there was a significant improvement in the atopic dermatitis, characterized by the absence of new skin lesions and subjective itching symptoms. Importantly, during the most recent follow-up, there has been no recurrence of atopic dermatitis. After this treatment, most of the affected areas showed hair regrowth, although some of the regenerated hair was white in color. The SALT score decreased to 20%. However, the patient experienced eyebrow loss during the treatment period (**Figure 3**). Discontinuation of Dupilumab and initiation of oral Baricitinib at a dose of 2mg once daily resulted in further improvement in hair regrowth after 5 months, the eyebrows and hair have essentially fully recovered. The patient is advised to gradually reduce the dosage (**Figure 4**).During the treatment, regular blood tests and tests assessing liver and kidney function were performed. The pre-treatment laboratory investigations revealed the following results: alanine aminotransferase (ALT) of 5 IU/L (reference range: 0-40 IU/L), aspartate aminotransferase (AST) of 21 IU/L (reference range: 0-42 IU/L), total bilirubin of 7.34 μmol/L (reference range: ≤23 μmol/L), direct bilirubin of 0.87 μmol/L (reference range: ≤8 μmol/L), serum total bile acids of 6.3 μmol/L (reference range: 0-10 μmol/L), uric acid of 277 μmol/L (reference range: 150-420 μmol/L), and creatinine of 48 μmol/L (reference range: 35-106 μmol/L). Following oral administration of baricitinib, the reevaluation of the aforementioned parameters showed the following results: ALT of 6 IU/L, AST of 26 IU/L, total bilirubin of 7.09 μmol/L direct bilirubin of 2.24 μmol/L, serum total bile acids of 4.9 μmol/L, uric acid of 274 μmol/L, and creatinine of 45 μmol/L. The pre-treatment hematological examination revealed the following results: total white blood cell count of 9.1 x 10^9/L (reference range: 3.5-9.5 x 10^9/L); neutrophil percentage of 44.6% (reference range: 40%-75%); lymphocyte percentage of 45.7% (reference range: 20%-50%); red blood cell count of 5.02 x 10^9/L (reference range: 3.8-5.1 x 10^9/L); hemoglobin level of 137 g/L (reference range: 115-150 g/L); and platelet count of 261 x 10^9/L (reference range: 125-350 x 10^9/L). Upon reevaluation after treatment, the white blood cell count remained unchanged at 9.1 x 10^9/L; neutrophil percentage was 44.6%, lymphocyte percentage was 45.7%; red blood cell count was 5.02 x 10^9/L, hemoglobin level was 137 g/L, and platelet count was 261 x 10^9/L. Immunoglobulin E (IgE) levels decreased. The IgE level before treatment was measured at 323 IU/mL(reference range: 5-161 IU/mL). After 3 months of treatment with Dupilumab, the IgE levels decreased to 277 IU/mL, and after 6 months of treatment, they further decreased to 140 IU/mL, within the normal range. Both dupilumab and baricitinib were prescribed off-label for this patient. After a thorough assessment of the patient’s condition and treatment requirements, and extensive communication with the patient’s caregivers, we implemented the treatment plan mentioned above with explicit informed consent. This approach yielded satisfactory therapeutic outcomes. No adverse drug reactions were observed during the course of treatment.

In clinical practice, relapse of alopecia areata has been observed in some patients after discontinuing oral baricitinib. Therefore, it is crucial for patients to adhere to medical advice and either continue their medication or gradually taper the dosage to ensure the efficacy of JAK inhibitors. Discontinuing medication without medical guidance should be avoided to prevent relapse. During follow-up, close monitoring of the child’s condition and potential adverse drug reactions will be conducted. We will provide guidance on gradually tapering the medication or transitioning to safer topical or systemic therapies to maintain treatment efficacy.

## Discussion

Alopecia Areata (AA) is a prevalent autoimmune disorder characterized by non-scarring alopecia, which can range from localized patches on the scalp to total hair loss on the entire body. In the US population, the lifetime incidence of alopecia areata (AA) is estimated to be 2%. A nationwide cohort study of US employer-sponsored insurance found that the prevalence of AA ranged from 0.199% to 0.222% between 2016 and 2019, with an incidence rate of 91.46 to 92.90 cases per 100,000 patient-years (3, 4). Partial alopecia areata can spontaneously resolve in some patients, but it is often characterized by a high risk of relapse. In certain cases, the condition can progress to severe forms such as alopecia totalis. AA often coexists with atopic dermatitis (AD), and the severity and susceptibility of the diseases are correlated. Treatment of AA can be challenging and significantly impacts the physical and mental well-being of patients. The prevalence of AA is higher in children than in adults, and children commonly experience psychological issues related to hair loss. Moreover, treatment options for pediatric AA are more limited compared to adults. Therefore, seeking safe and effective treatments for pediatric AA is crucial (5). A cohort study conducted in the UK suggests that patients with Alopecia Areata are at a higher risk of developing autoimmune diseases, including rheumatoid arthritis, systemic lupus erythematosus, psoriasis, and atopic dermatitis, among others. In particular, the prevalence of atopic dermatitis in the Alopecia Areata group was found to be 15.7%, while it was only 7.8% in the control group (6).The pathogenesis of AA is not fully understood, and it may involve heterogeneity. Some studies suggest that interferon-gamma (IFN-γ)-mediated Th1 response induces AA, while others indicate that Th2-mediated immune response and Treg cell deficiency play a major role in AA development (7, 8). The JAK/STAT signaling pathway is involved in various important biological processes, including cell proliferation, differentiation, apoptosis, and immune regulation, through the signaling of multiple cytokines and growth factors. Several cytokine pathways implicated in AA pathogenesis, such as IFN-γ and the common gamma chain cytokine family, can participate in AA development via the JAK/STAT pathway. Research also suggests that certain cytokines, such as IL-4, enhance Th2 cell differentiation through the JAK pathway and contribute to the inflammatory immune response in AD (9, 10).

Baricitinib, a selective JAK1/JAK2 inhibitor, is the first targeted therapy approved by the FDA for systemic treatment of alopecia areata (AA). Clinical trials in phase II and III have confirmed the effectiveness and safety of baricitinib in treating severe AA in adults (11–13). Currently, literature data on the use of JAK inhibitors for treating pediatric AA are based on individual case reports and case series. However, previous studies have indicated that the JAK/STAT signaling pathway is a key target for treating AA and AD. There have been relevant case reports demonstrating the effectiveness and safety of JAK inhibitors in these patients. Therefore, for children with both conditions, baricitinib is one of the available treatment options (14).

Atopic dermatitis is a chronic, non-infectious inflammatory skin disease characterized by persistent itching. It commonly presents with eczematous skin lesions, including erythema and papules. This condition is more prevalent in children (15). A meta-analysis has confirmed that alopecia areata significantly raises the risk of atopic dermatitis, particularly in cases of early-onset alopecia areata (before 10-13 years of age) and severe alopecia areata (16). Alopecia areata is considered a type 1 inflammatory disease, where activated NKG2D+CD8+ cells produce the Th1 cytokine interferon-gamma. This disrupts immune tolerance of hair follicles and exposes self-antigens, resulting in dense infiltration of inflammatory cells and apoptosis around the hair follicles, ultimately leading to hair loss (7). It is widely accepted that atopic dermatitis (AD) is primarily driven by Th2 immune responses. A meta-analysis has indicated a potential correlation between the severity of alopecia areata (AA) and AD, with worse outcomes observed in patients with concurrent onset of both conditions. While both AD and AA are classified as inflammatory disorders, the clinical association between the two may not be fully explained by the traditional Th1/Th2 paradigm. Nevertheless, recent research has demonstrated a Th2 cytokine bias in AA. Elevated levels of Th2 cytokines (such as IL-4, IL-5, IL-6), IgE, and eosinophils have been observed in the serum of AA patients, suggesting that Th2 bias may play a significant role in the pathogenesis of AA (17–19).

Dupilumab has been approved for the treatment of moderate to severe atopic dermatitis (AD) in children, as well as in adults. Theoretically, downregulating the Th2 pathway through treatment with dupilumab may enhance the Th1 pathway, which could potentially induce or exacerbate alopecia areata (AA). There have been clinical reports documenting similar cases in which AA was observed or worsened after treatment (20) (19). It is interesting to note that in clinical practice, patients with both alopecia areata (AA) and atopic dermatitis (AD) have shown improvements in symptoms of both conditions after receiving treatment with dupilumab. The child described in this report experienced a reduction in AA and achieved hair regrowth in certain areas affected by hair loss following dupilumab treatment (21). In a clinical study, patients with alopecia areata (AA) with or without atopic dermatitis (AD) were randomly assigned in a 2:1 ratio to receive treatment with dupilumab injections or placebo. After 24 weeks of treatment, it was observed that the placebo group experienced further progression of the disease, while the dupilumab group showed improvement in SALT scores. Furthermore, in patients with IgE levels ≥200IU/mL, the rate of remission further increased after 48 weeks of treatment, suggesting the involvement of Th2-mediated inflammatory response in the pathogenesis of AA. Additionally, baseline IgE levels may be considered as one of the predictive indicators of dupilumab efficacy (22). It is worth noting that in this particular case, the initial IgE levels of the patient were elevated. After treatment, there was an improvement in both hair loss and skin itching symptoms, along with a decrease in serum IgE levels. This further confirms the previously mentioned conclusion.

In this report, the patient also experienced hair regrowth in some areas of alopecia after treatment with Dupilumab, which may indicate that Dupilumab has some potential for treating AA. However, there was eyebrow loss. In the case of this patient, it is important to recognize that she had severe alopecia areata (AA) with extensive hair loss and a prolonged duration of the disease. The effectiveness of using Dupilumab as a standalone treatment for severe alopecia areata is limited. The patient’s eyebrow hair loss further indicates that Dupilumab alone has not fully stopped the immune system-mediated damage to the hair follicles. Therefore, it is necessary to consider altering the treatment plan. After an additional 5 months of oral treatment with baricitinib, the SALT score decreased to less than 10%.

The patient in this case was diagnosed with severe AA in combination with AD, with a long disease duration and poor response to local treatment. However, with the sequentially therapy, the patient showed significant improvement, suggesting that the sequential treatment of dupilumab and baricitinib may hold potential for severe AA combined with AD. Long-term follow-up of this patient is necessary, and a large number of multicenter, double-blind, randomized controlled trials are needed to demonstrate the long-term efficacy and safety of dupilumab and baricitinib treatment. This will greatly facilitate the application of sequentially therapy in patients with alopecia areata and atopic dermatitis, benefiting a large number of patients.

## Data availability statement

The original contributions presented in the study are included in the article/supplementary material. Further inquiries can be directed to the corresponding author.

## Ethics statement

Written informed consent was obtained from the individual(s), and minor(s)’ legal guardian/next of kin, for the publication of any potentially identifiable images or data included in this article. The studies involving human/animal participants were reviewed and approved by Chinese-Japanese Friendship Hospital Clinical Research Ethics Committee.

## Author contributions

HF: Writing – original draft, Conceptualization, Investigation, Writing – review & editing. FZ: Conceptualization, Writing – original draft. WL: Investigation, Writing – original draft. YJ: Data curation, Writing – original draft. QL: Investigation, Writing – original draft. DY: Writing – review & editing, Writing – original draft.
